# Complement Activation by Merozoite Antigens of *Plasmodium falciparum*

**DOI:** 10.1371/journal.pone.0105093

**Published:** 2014-08-21

**Authors:** Jackson C. Korir, Nancy K. Nyakoe, George Awinda, John N. Waitumbi

**Affiliations:** 1 Masinde Muliro University of Science and Technology, Kakamega, Kenya; 2 Walter Reed Project, Kenya Medical Research Institute, Kisumu, Kenya; Kenya Medical Research Institute (KEMRI), Kenya

## Abstract

**Background:**

Complement (C) is a crucial part of the innate immune system and becomes over activated during malaria, resulting in depletion of C components, especially those for lectin pathway (LP), thereby compromising the host's innate defense. In this study, involvement of *P. falciparum* antigens in C activation was investigated.

**Methods:**

A highly synchronous culture of the Dd2 clone of *P. falciparum* was established in a serum free medium. Supernatants harvested from rings, trophozoites and schizonts at various parasite densities were tested for ability to activate C by quantifying amount of C3b deposited on erythrocytes (E). Uninfected sham culture was used as control. Remnants of each C pathway were determined using Wieslab complement System Screenkit (Euro-diagnostica, Sweden). To identify MBL binding antigens of LP, culture supernatants were added to MBL sepharose columns and trapped antigens eluted with increasing concentrations of EDTA (10 mM, 50 mM and 100 mM) and then desalted before being tested for ability to activate C. The EDTA eluate with highest activity was run on a polyacrylamide gel and silver stained proteins analyzed by mass spectroscopy.

**Results:**

Antigens released by *P. falciparum* growing in culture activated C leading to C3b deposition on E. Maximal activation at 7% parasitemia was associated with schizont stage (36.7%) compared to 22% for rings, 21% for trophozoites and 3% for sham culture. All the three pathways of C were activated, with highest activation being for the alternative pathway (only 6% of C activation potential remained), 65% for classiical and 43% for the LP. Seven MBL binding merozoite proteins were identified by mass spectrometry in the 50 mM EDTA eluate.

**Conclusions:**

MBL binding merozoite adhesins with ability to activate C pathway were identified. The survival advantage for such pronounced C activation is unclear, but opsonisation could facilitate recognition and invasion of E.

## Background

That infection with malaria parasites is associated with increased complement (C) activation has been known for decades [1]. Several molecules have been incriminated in C activation: some expressed on the surface of infected erythrocytes (iE), others are released following schizont rupture, or are part of circulating immune complexes (IC) [1], [2], [3]. Studies by Roestenberg *et al.* demonstrated that intact or lysed *P. falciparum* iE are capable of stimulating the plasma of malaria-naïve individuals leading to the formation of terminal C complex [4]. It was hypothesized that iE are able to regulate C activation with the use of erythrocyte-bound C regulatory proteins, and in this way, elicit only limited amounts of terminal C activation products. However, with destruction of the iE, degradation products are released, generating higher levels of terminal C complex [4]. The breakdown products of iE such as hemozoin and hematin, have been shown to possess strong pro-inflammatory properties that can activate C [5], [6].

Activation of all three pathways of the C cascade has been demonstrated during malaria infection. A case control study in Kenya showed excessive activation and consumption of C components during malaria, and the activation was dependent on malaria severity [7]. In that study, the activities of all three pathways of C were greatly reduced. Only 0.1% of lectin pathway (LP) was left (100% consumption), 10% for Classical Pathway (CP) (90% consumption) and 37% of Alternative Pathway (AP) (63% consumption). Previous studies had shown high plasma levels of spent C components of both the AP and CP in severe malaria [8]. It was later shown that purified hematin activates the AP promoting deposition of C3 breakdown products on E [6]. Hematin is released during intravascular hemolysis of iE and thus it was postulated that after multiple cycles of infection, the accumulating C3 breakdown products such as C3dg may be most efficiently bound by young E with the highest level of complement receptor-1 (CR1) and the resultant opsonization leads to their premature clearance [6].

Still, other studies have implicated the lectin pathway as being the most active in malaria and MBL has been shown to bind to iE during infection [9], [10]. The C binding domain of the MBL was shown to recognize the carbohydrate glycosylphosphatidylinositol (GPI) anchors of *P. falciparum* which are synthesized in a maturation-dependent manner, with schizonts and merozoites expressing most of GPI. Other studies have demonstrated recognition of glycosylated, immunogenic components of *P. falciparum* by MBL [10]. The importance of MBL in malaria may be inferred from a study that showed that children who have MBL deficiencies suffer from severe malaria, indicating that this pathway is important in controlling *P. falciparum* infection [11]. Still, other studies have shown ability of IC formed during malaria to activate the CP [1]. This study aimed at identifying malaria parasite molecules involved in C activation via the lectin pathway. We hypothesized that *P. falciparum* exploits these molecules to interact with innate immunity, perhaps as a survival strategy.

## Materials and Methods

### Ethics statement

Human blood used for culture of malaria parasites was obtained from volunteers who gave written consent under a study protocol that was approved by the Ethical Review Committee of the Kenya Medical Research Institute (SSC #, 1300) and the Walter Reed Army Institute of Research Human Subject Protection Board (WRAIR HSPB # 1919).

### *P. falciparum* culture

A Dd2 clone of *P. falciparum* obtained from cryo-preserved stock at the Walter Reed Project/KEMRI, Kisumu was used to initiate and maintain a parasite culture, essentially as described by Trager and Jensen, [12] with slight modifications. Briefly, the Dd2 parasites were thawed, washed and cultivated in washed group O^+^ human E diluted to 5% haematocrit in serum free RPMI 1640 media. Serum free RPMI 1640 media was prepared as described in the Malaria Research and Reference Reagent Resource (MR4) data sheet (MRA-156, MR4, ATCC Manassas Virginia) and the method of Schuster, 2002 [13]. Briefly, to 900 mL of tissue culture grade water was added to 10.43 g of powdered RPMI-1640 (without NaHCO_3_ and without L-glutamine). To this was added 25 mL of 1 M HEPES (final  = 25 mM), 26.7 mL of 7.5% sodium bicarbonate solution (final  = 0.2% NaHCO_3_), 10 mL of 200 mM L-glutamine (final  = 2 mM), 10 mL of 20% Glucose (final  = 20 mM), 10 mL of 0.5 mcg/mL hypoxanthine (final  = 0.005 mcg/mL), 0.25 mL of 10 mg/mL Gentamicin (final  = 2.5 mcg/mL), and 0.5% (w/v) AlbuMax II (final of 5 g/L). Tissue culture grade water was then added to make 1.0 L and mixed thoroughly. The pH was adjusted to 6.8 and then filtered with 0.22 micron Millipore filter unit. All the culture reagents were from Sigma-Aldrich, Missouri, USA. As control, a ‘sham’ culture was established in conditions similar to those of parasite culture except that there were no malaria parasites. The growth and development of the malaria parasites was monitored through examination of blood smears by microscopy. Synchronization of malaria parasites was performed by enriching for young ring stage trophozoites using 5% D-sorbitol which selectively destroys multinucleate iE [14]. Briefly, asynchronous culture with parasites at early trophozoites was centrifuged at 1620 *xg* for 5 minutes and the size of pellet estimated. For every 100 micro liter of the pellet, 2 mL of 5% sorbitol was added, mixed and incubated at 25°C for 10 minutes. Thereafter, the cell suspension was spun at 1620 *xg* for 5 minutes followed by aspiration of the supernatant. The cells were then washed two times with RPMI 1640 media and after suspension of the remaining pellet, the malaria parasites were grown in culture as previously described. Synchronization was repeated twice after every growth cycle (approximately 48 hours). Culture supernatants from nearly 100% rings, late trophozoites and schizonts were collected by centrifuging at 1620 *xg* for 5 minutes at different parasitemias.

### Complement activation by malaria culture supernatant

Supernatant collected from the rings, trophozoites and schizonts stages as well as from the sham culture were tested for their ability to activate C by quantifying amount of C3b deposited on E. Normal uninfected Es were washed three times with PBS by centrifugation at 1620 *xg* for 5 minutes and re-suspended at 1% by adding 10 micro liters of the E suspension to 990 micro liters of PBS. 10 micro liters of 1% E (equivalent to 10^6^ E) were then added to 15 micro liters of AB+ serum, followed by addition of 15 micro liters of the culture supernatant. The cell mix was gently vortexed and the tubes were incubated at 37°C for 10 minutes, washed twice in 1 mL 1% BSA in PBS by centrifugation at 1620 *xg* for 3 minutes. The resulting pellet was then re-suspended in 50 micro liter solution of 1% BSA/PBS and 50 micro liter of anti-C3b antibody (clone 3E7, a gift from Prof Ronald P. Taylor, University of Virginia, Charlottesville, USA) conjugated to Alexa 488 at 1 mcg/mL. The suspension was incubated for 10 minutes at 37°C, washed twice in 1% BSA/PBS and then re-suspended in 1% PFA. Deposited C3b was quantified on a BD FACSCAN cytometer using CellQuest pro version 2. Es were gated based on the forward and side scatter on a logarithmic scale and 10,000 events acquired. The data was then analyzed with FlowJO version 8.0 software (Tree Star Inc., Ashland, USA). The remaining Es were centrifuged with a CytopSpin Shandon 4 (Thermoscientific, Massachusetts, USA) onto a high binding slide at speed of 500 rpm for 5 minutes. The cells were observed under high power magnification using an Olympus BX41 fluorescent microscope (Olympus America Inc. USA) using the green filter.

### Determination of complement activation potential of malaria culture supernatant

Culture supernatant or sham was used to activate C. For this, 50 micro liters of culture supernatant or sham harvested at 7% was added to 50 micro liters AB^+^ serum and the reaction mix incubated at 37°C for 10 minutes to allow C activation to occur. The remnant C for each pathway was determined using Wieslab Complement System Screenkits (Euro-diagnostica, Malmo, Sweden) essentially as described previously [7], [15], [16]. The kits are supplied pre-coated with specific activators for the CP, LP and AP. To measure the remnant C activity for each pathway, the reaction mix was diluted into buffers supplied in the Wieslab Complement System Screenkits that contained specific blockers to ensure that only the specific C pathway was activated. For measurements of CP and LP, the samples were diluted at 1∶100, and 1∶18 for the AP. For quality control of the assay and for calculation of the functional activities, positive and negative controls with known activities were used as recommended by the kit manufacturer. Briefly, 100 micro liters of each diluted sample and control were added in duplicate to the respective pathway-specific wells and the plate incubated for 70 min at 37°C. Wells were then aspirated and washed thrice with wash buffer. Then, 100 micro liters of conjugate containing alkaline phosphatase-labeled antibodies to C5b-9 was added to each well and incubated for 30 min at 25°C. Wells were again washed thrice and 100 micro liters of substrate [(3,3′,5,5′ tetramethylbenzidine (TMB)] added and the samples were incubated for 30 min at 25°C to allow for color development. Absorbance was immediately read at 405 nm. Functional activity for the respective pathways was then calculated using the OD values of the positive and negative controls provided in the kit: OD Sample – OD Negative Control/(OD Positive Control – OD Negative Control)*100. Presence of all pathways of complement in the AB serum was also measured before activation.

### Identification of *P. falciparum* antigens that bind MBL

1 mL of 90% pure MBL (Statens Serum Institute, Denmark) was first dialyzed against coupling buffer (0.1 M NaHCO3, 0.5 M NaCl, pH 9.0) for 1 hour. The pre-coupling OD_280_ of the MBL solution was read. The purified MBL was coupled to 1 g of Cyanogen Bromide Sepharose beads (Amersham Pharmacia, New Jersey, USA) that had been swollen in 1 mM HCl for 15 min at 25°C. The swollen gel slurry was transferred into a sintered glass funnel and washed with 200 mL of 2 mM HCl, followed by a wash in coupling buffer. The washed gel was transferred into a polystyrene tube containing the purified MBL solution and the tube rotated gently for 2 hours at 25°C. The gel was then centrifuged for 1 minute at 1500 *xg* and OD_280_ of the supernatant read and compared to the initial reading. Coupling was considered successful if the second OD_280_ reading was ten-fold lower than the initial reading of the purified MBL alone. The gel was then washed three times in coupling buffer and incubated with blocking buffer (1 M Tris-Base, pH 9.0 in coupling buffer) for two hours at 25°C. The purpose of blocking was to ensure that the binding sites of the sepharose were all occupied so that the sample may not bind directly to the sepharose but only to the MBL. The coupled beads were then washed 4x, alternating between low pH wash buffers (0.1 M acetic acid, 0.5 M NaCl) and coupling buffer and checking OD_280_ of first and fourth supernatants to ensure that the OD_280_ readings were minimal (0.01 or lower) to confirm that no detaching of the coupled MBL was taking place. The gel was then poured into column and washed with 5 column volumes of sterile-filtered PBS-azide and stored at 4°C in sterile-filtered PBS-azide until used.

Culture supernatant (1 mL) was pre- equilibrated with an equal volume of TBS/2 mM CaCl_2_/0.2% Tween and allowed to warm up to at 25°C. The equilibrated supernatant was then allowed to run through the MBL column, followed by two washes with plain PBS. The proteins that bound to the MBL-sepharose column were then eluted using 2.5 mM of TBS containing increasing concentrations of EDTA (10, 25, 50 and 100 mM) [17]. Each of the eluted fractions was collected separately in a sterile polystyrene tube. EDTA was then removed in desalting columns (GE healthcare, Sweden) as recommended by the manufacturers in 3.5 mL distilled water. The fractions were then vacuum concentrated by lyophilisation using a refrigerated CentriVap Vacuum Concentrator (Labconco Corporation, Missouri, USA) and stored at −80°C. Just before use, the lypophilized samples were re-suspended to the original culture volume (1 mL in PBS).

To test for ability of the eluted fractions to activate C, flow cytometric analyses were performed as detailed in the section entitled “**Complement activation by malaria culture supernatant”** with the substitution of the malaria culture supernatant by the eluted fractions.

### Polyacrylamide Gel Electrophoresis and Mass Spectroscopic analysis of MBL binding antigens

To 6 micro liters of the desalted MBL eluate was added to 3.75 micro liters of 4X Lithium Dodecyl Sulfate (LDS) sample buffer and then loaded into wells of NuPAGE Novex Bis-Tris 4–12% pre-cast gel (Invitrogen Corporation, Carlsbad, USA). A pre-stained standard weight marker (Sea Blue Plus2 from Invitrogen Corporation) was also loaded. After assembly into electrophoretic tank, the proteins were electrophoresed at 200 V for 50 minutes and the gel kept overnight at 4°C before staining with silver (GelCode SilverSNAP stain kit II, Pierce, Rockford, IL, USA). The stained protein bands were removed from the gel and sent to Ruprecht-Karls-University, Heldelberg, Germany for Matrix-assisted laser desorption/ionization (MALDI). Peptide reports were generated and used to query the SwissProt data base.

## Results

### Complement activation by *P. falciparum* culture supernatant

**Figure 1:**
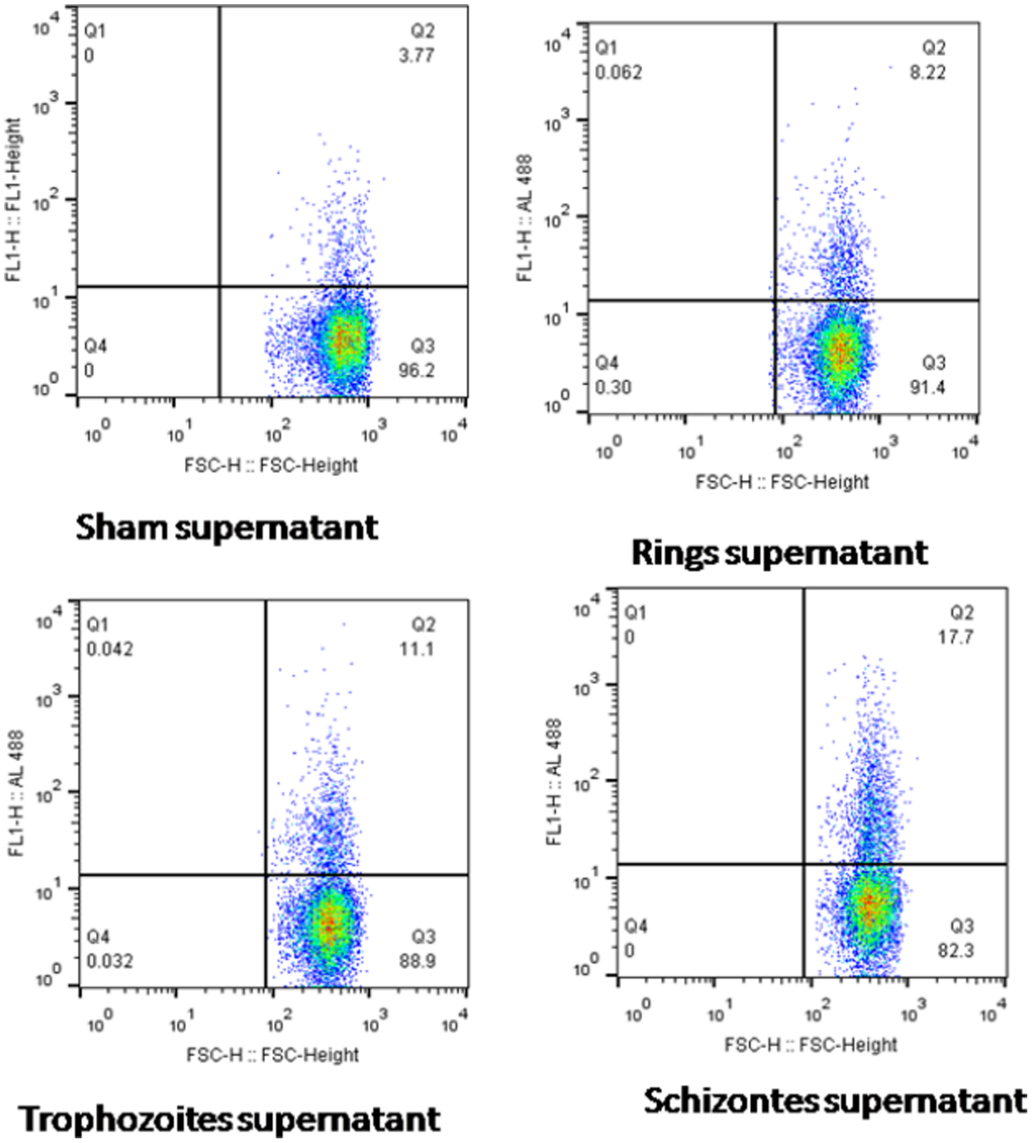
Representative dot plots showing % C3b deposition on RBC following complement activation by sham and malaria culture supernatants at 2% parasitemia. % C3b deposition for sham supernatant was (3.77%), rings (8.22%), late trophozoites (11.10%) and schizontes (17.70%).

Flow cytometric analysis showing C3b deposition on E by culture supernatant collected from the three developmental stages of *P*. *falciparum* are shown in Figure 1. The amount deposited was stage dependent, being lowest for early trophozoites (8.22%), 11.10% for late trophozoites and highest for schizontes (17.70%). This observation is consistent with previous finding that have shown various malaria parasite products including hemozoin, hematin and GPI activate complement [1], [5], [6], [11].

**Figure 2:**
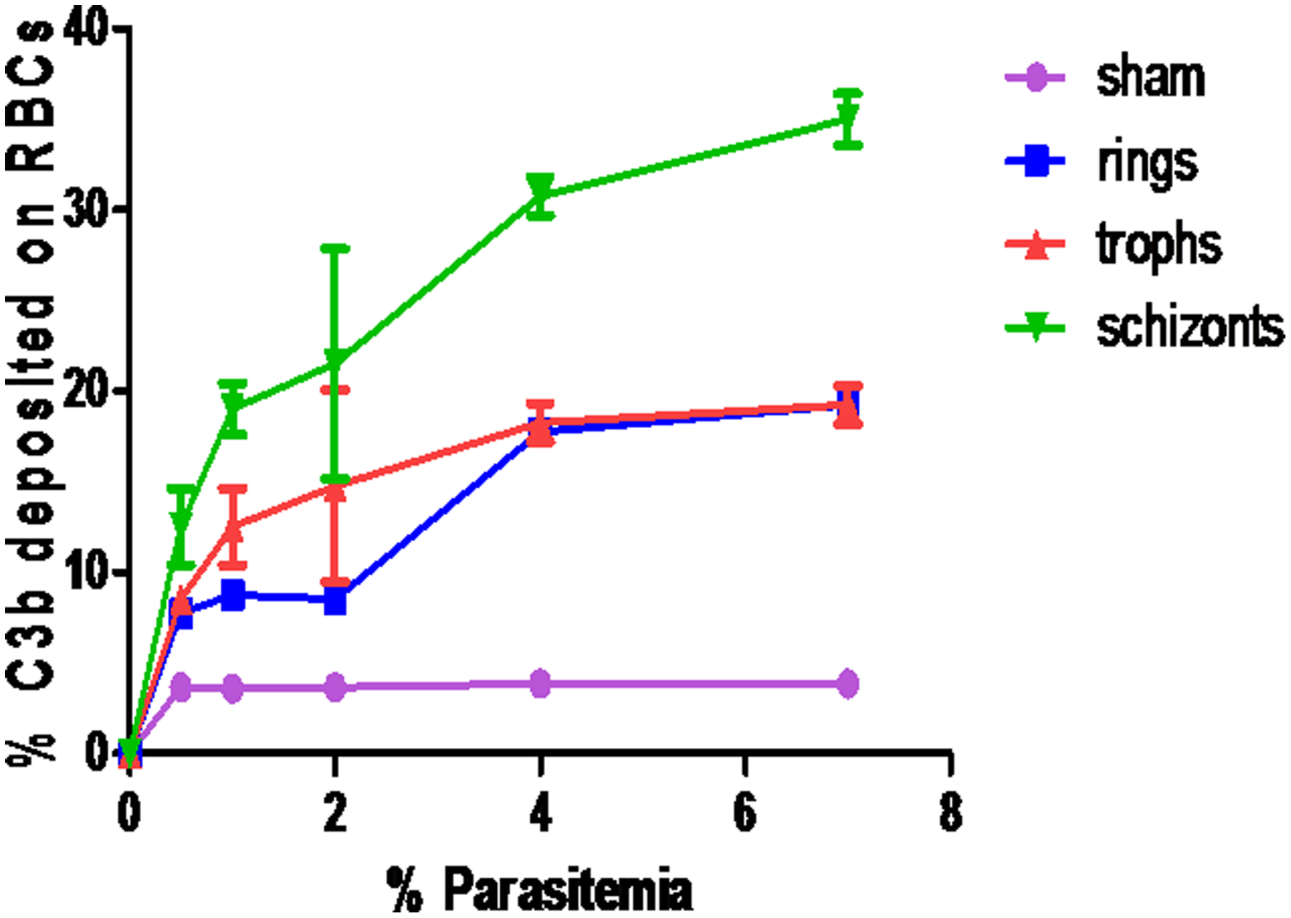
Complement activation by culture supernatant collected from different growth stages and parasite densities of *P. falciparum*. At all parasitemia levels, % C3b deposition was highest with supernatant harvested at schizont stage.

**Figure 3:**
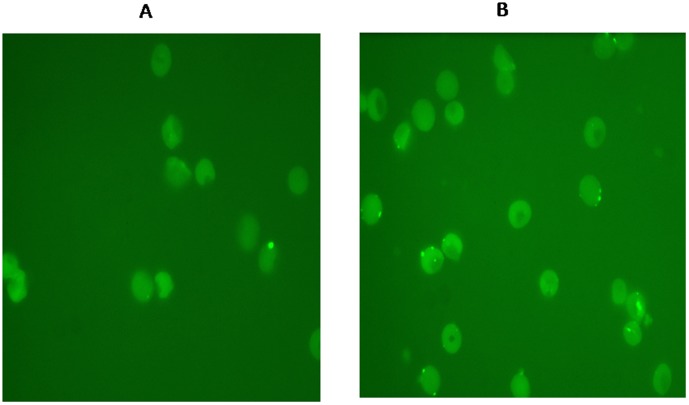
Photomicrographs showing C3b deposition on erythrocytes (fluorescent spots) following activation of complement by supernatant from sham culture (Panel A) and *P. falciparum* culture (Panel B) harvested at schizont stage. Note the fluorescent spots are in clusters, which is the hallmark of CR1 distribution on erythrocytes [33]. C3b was detected by Alexa 488 conjugated to anti-C3b IgG, and observed under high power magnification using an Olympus BX41 fluorescent microscope (Olympus America Inc. USA).

As shown in Figure 2, the amount C3b deposited was also dependent on parasitemia, with the schizont stage having a mean of 19.76% (6.52–33.06 CI) at 7% parasitemia, 12.21% (4.70–19.72 CI) for the trophozoites and 10.33% (2.80–17.81 CI) for the ring stages, at the same parasitemia. Supernatant from the sham culture did not activate complement. On fluorescent microscope, the C3b deposition (fluorescent spots), were in clusters that bear the hallmark of CR1 on E (Figure 3).

### *P. falciparum* culture supernatant activates all the three pathways of the complement

**Figure 4:**
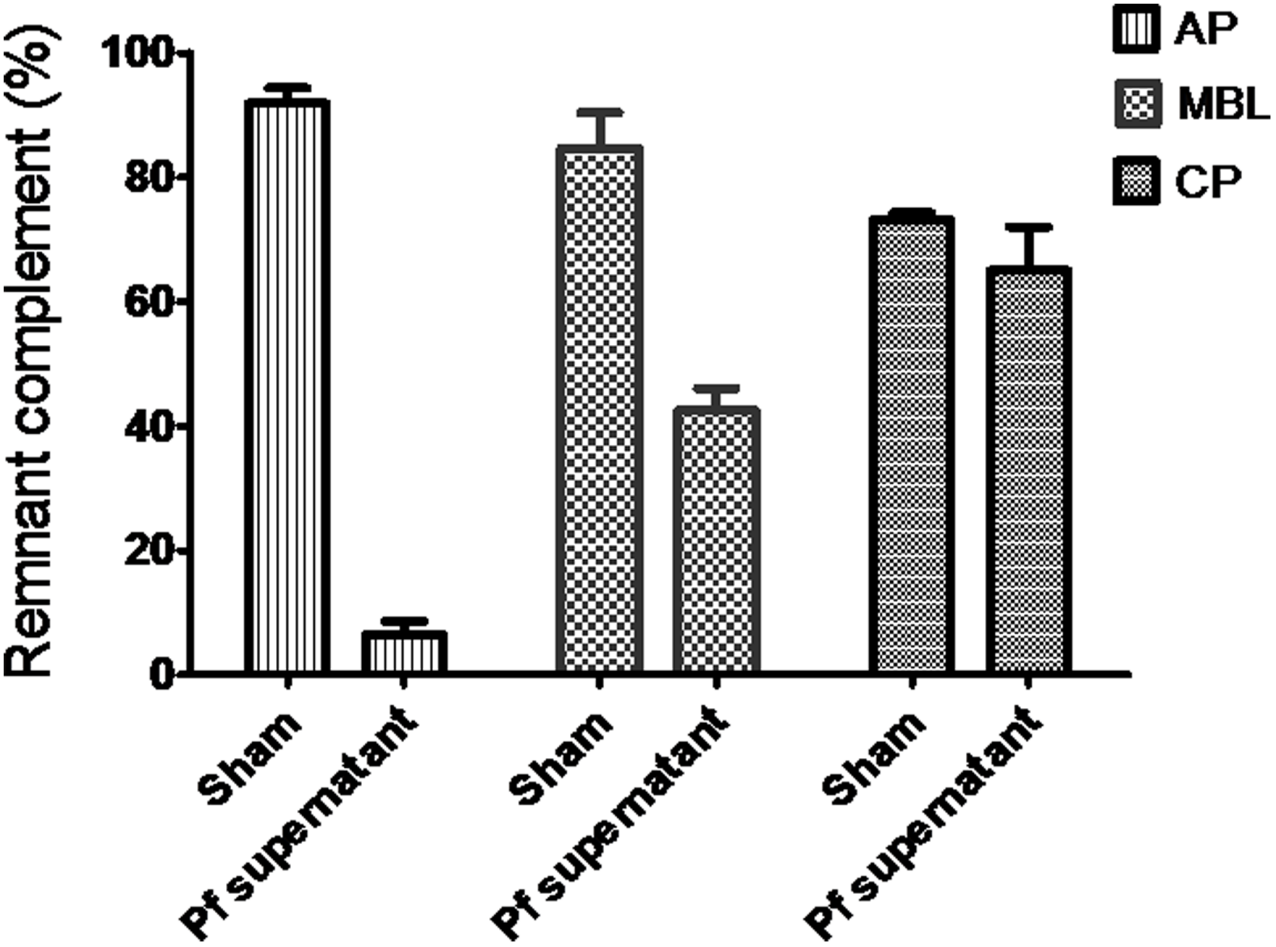
Remnant complement components of AP (6%), MBL (43%) and CP (65%) following activation by *P. falciparum* culture supernatant. Sham supernatant did not lead to depletion of complement components.

Before complement activation, activation potential for the AB serum was determined to be 94% for AP, 82.5% CP and 91.5% for LP. This was important, especially for the LP, because the gene for human MBL, called *MBL2*, is polymorphic at coding regions, at promoter and 5′ -untranslated regions. Depending on the mutation, certain genotypes may affect circulating antigen levels and/or functional activity. It is thus possible to have high MBL levels that are not correlated with functional activity. It is for this reason that Wieslab complement System Screen kit that assesses complement function as opposed to antigen levels was used. As shown in Figure 4, on activation by malaria culture supernatant, the complement activation potential for the AB serum reduced from 94% to 6% for AP, 91.5% to 43% for LP and 82.5% to 65% for CP. Sham supernatant had minimal ability to activate complement.

### Identification of *P. falciparum* lectin binding proteins that activate complement

**Figure 5:**
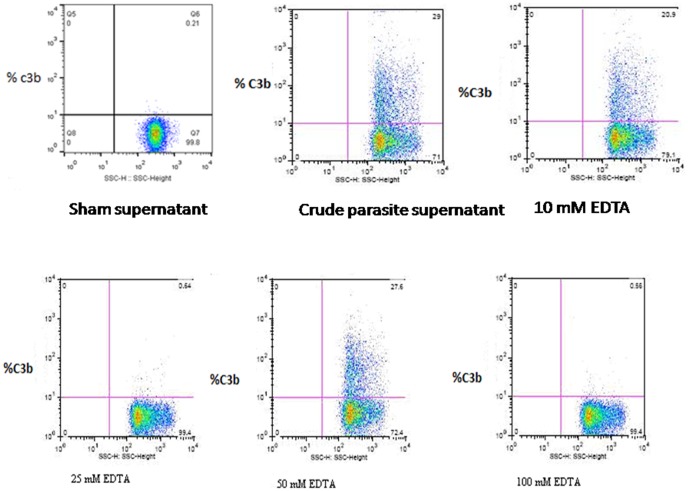
Dot plots showing complement activation by fractions eluted at different EDTA concentrations. The 50 mM fraction (Panel D) had maximal activity that was comparable to that of the crude culture supernatant (Panel A). Sham supernatant eluted at 50 mM EDTA had no complement activity.

Figure 5 shows amount of C3b deposited on E following C activation by fractions eluted from MBL column compared to crude malaria culture supernatant. Maximal activity was observed in the fraction eluted with 50 mM EDTA (28% vs 29% by crude extract), compared to 21% by 10 mM EDTA, 0% by 25 mM and 100 mM EDTA. Sham supernatant eluted at 50 mM EDTA had no complement activity.

**Figure 6:**
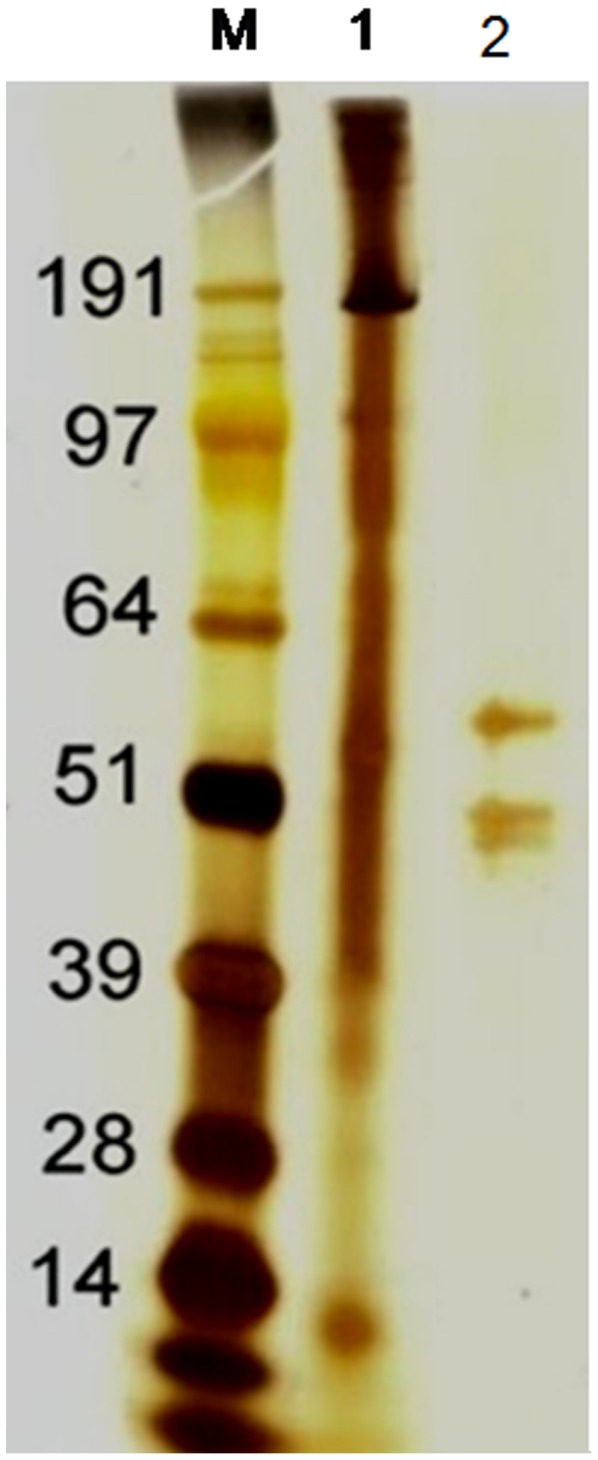
Silver stained PAGE gel showing three distinct proteins of sizes 40–64 kDa. Lanes marked MW =  molecular weight marker, 1 =  crude *P. falciparum* culture supernatant, 2 =  MBL binding proteins eluted with 50 mM EDTA.

Since the 50 mM fraction had comparable activity to that of the crude malaria culture supernatant, it was processed further to allow for identification of malaria antigens that activate C. Figure 6 shows silver stained gel with three proteins of between 40 kDa to 64 kDa. On mass spectrometry, 7 merozoite proteins were identified (Table 1). The detected proteins had predicted MWs much greater than that observed in the SDS-PAGE, probably as a result of proteolytic processing.

**Table 1:** Merozoite lectin binding proteins identified by Matrix-assisted laser desorption/ionization (MALDI).

Protein name	Gene bank accession #	Molecular weight (Da)
Reticulocyte-binding protein homologue 1	PFD0110w	357,823
Reticulocyte-binding protein 2 homolog a	PF13_0198 PE	370,436
LRR domain-containing protein	PFC0760c	402,952
MATH and LRR domain-containing protein	PFE0570w	1,187,585
Uncharacterized protein	MAL13P1.304	210,164
Uncharacterized protein	PF11_0213	303,407
Uncharacterized protein	PFB0765w	167,008

## Discussion

It has been known for decades that infection with malaria parasites is associated with increased C activation [1] that leads to uncompensated consumption of all the three pathways of C [7]. In this study, an *in vitro* culture system was used to further elucidate the role of malaria antigens in C activation. Spent *P. falciparum* culture supernatant was able to activate C (Figures 1 and 2) and the activation was stage and parasite density dependent. The supernatant harvested following schizont rapture had maximal ability to activate C, implying involvement of merozoite antigens or other molecules released subsequent to rapture of iEs. Potential molecules include hemozoin/hematin [5], [6], GPI [9], [10], [11], antigens that exist as IC [1], [18], [19], merozoite antigens and probably many others that are still unknown.

Neva and colleagues found C activation occurred during or soon after schizont rupture and seemed to be dependent on the parasite density and presence of C fixing antibodies [20]. In the current study, activation of the C increased as the amount of supernatant used increased and then declined probably due to diminishing amounts of the C components, confirming presence of factors in the culture supernatant that have ability to activate C (Figure 2).

Following C activation, the remnant components of the different pathways of the cascade were tested to determine levels of activation of the different pathways. As shown in Figure 4, all pathways were activated, albeit to different levels, with AP being the most activated (only 6% remained), compared to 65% for CP and 43% for LP, indicating the diversity of C activating molecules present in the culture supernatants. Some of the excessive activation of AP could have come from direct activation of CP and/or LP through the amplification loop. Hemoglobin degradation products specifically hemozoin and hematin have been shown to preferentially activate AP [6]. Activation of CP is commonly observed in patient with malaria [7], [8] and has been attributed to presence of IC [1], molecules on the surface of iE [21], as well as malaria DNA fragments [22]. In absence of IC in the malaria culture system used in the current study, activation of CP is likely due to malaria parasites molecules.

The LP pathway is typically activated through the binding of mannose. A study by Garred et al [9] showed that MBL could interact with the GPI anchors of *P. falciparum* which are primarily expressed by schizonts and merozoites. Later studies demonstrated the ability of glycosylated, immunogenic components of *P. falciparum* to interact with MBL [23]. Previous work has showed that children with severe malarial anemia over activate the LP leading to depletion of this innate pathway of the host defence [7]. In the current study, MBL column was used to isolate malaria parasites proteins that ligate to MBL. Table 1 shows seven merozoites proteins that were isolated through this process. Two of these proteins, the reticulocyte-binding protein and reticulocyte-binding protein 2 homologue are adhesins expressed by merozoites and have been shown to be involved in invasion of E [24], [25]. The LRR domain-containing protein (Gene bank accession # PFCO760c) is a conserved *Plasmodium* protein of unknown function [26]. The MATH and LRR domain-containing protein is thought to be involved in the invasion E [26], as well as the growth and survival of malaria parasites during development within the Es. The other three, MAL13P1.304, PF11_0213 and PFB0765w have no known function. As shown in Table 1, the detected proteins had predicted MWs much greater than 40–64 kDa observed in the SDS-PAGE. One possible explanation is that these proteins exist as much larger proteins that become cleaved by proteolysis. This is at least known to occur for the RH family that are differentially processed in the schizont and during invasion, resulting in proteins of different sizes with different erythrocyte binding properties [27].

There is no evidence to indicate that all the identified proteins specifically interact with MBL. Since most of these proteins are “sticky”, they could stick to other proteins immobilised on sepharose beads. Proteins in the RH family are ‘sticky’ and could interact non-specifically with other proteins. Further experiments will be needed to identify the specific proteins that interact with MBL to cause complement activation. Also, none of the identified merozoite proteins are predicted to have GPI anchor that is known to interact with MBL [28]. Because we only examined proteins that eluted at 50 mM EDTA, we may have missed other MBL binding proteins.

During invasion, merozoite adhesins localize to the apical tip of the merozoite and interact with specific host receptors to initiate parasite entry [29]. A number of studies show that *P. falciparum* uses a key functional site in C receptor type-1 (CR1) for invasion of human Es [30], [31], [32]. CR1 is also the receptor for C3b, and as shown in Figure 3, the clustering of C3b deposition bears the hallmark of CR1 clustered distribution on the E [33]. It's unclear why merozoite proteins would cause such pronounced activation of complement. It's tempting to speculate that merozoites could potentially be using these adhesins to activate C, and the subsequent C3b opsonisation serves as a strategy for recognition and invasion of Es. Further studies are needed prove these assertions.
